# Adrenal schwannoma: a case report

**DOI:** 10.1259/bjrcr.20190044

**Published:** 2020-02-12

**Authors:** Giuseppina Dell'Aversano Orabona, Domenico Ricci, Ilaria Emili, Francesca Serpi, Valerio Ferrara, Angelo Vanzulli

**Affiliations:** 1 Department of Advanced Biomedical Sciences, "Federico II" University of Naples, Via S. Pansini, 5, 81031, Naples, Italy; 2 Department of Biomedical Sciences for Health, University of Milan –Via Carlo Pascal 35, 20133, Milan, Italy; 3 Department of Oncology and Hemato-oncology, University of Milan, Via Carlo Pascal 35, 20133, Milan, Italy; 4 Department of Advanced Technologies, ASST Grande Ospedale Metropolitano Niguarda,, Milan, Italy

## Abstract

Retroperitoneum is an uncommon site for Schwannoma tumors and among adrenal incidentaloma the Schwannoma is rare. This condition lacks of a specific clinical and radiological features, but correct diagnosis before pathological examination should be very important for clinical management and surgical decision. We describe a case of voluminous retroperitoneal incidental mass, that was proved to be an adrenal Schwannoma.

## Introduction

Shwannoma, also called neurilemmoma, is a tumor with very low incidence (<1%) of malignant changes and derives from the myelinic sheath of peripheral autonomic or cranial nerves. Schwannomas arise from Schwann cells, so they can appear in any portion of neural tissue where cells of Schwann are present but they prefer neck and head regions or the flexor surfaces of the upper and lower extremities.^1,2^ All other sites are rare (<10% of cases)^3,4^ and only a few retroperitoneal cases have been described in literature.^4–12^ However, retroperitoneal Schwannomas are often large size lesions with tendency to displace, and more rarely to invade, surrounding structures before they become clinical apparent.^13^ Consequently, visceral and adrenal Schwannoma are usually discovered incidentally.^14^ In a review of 3979 adrenal lesions, only 19 patients were effectively affected by adrenal Schwannoma (0.48%)^15^ and approximately 40 cases of adrenal Shwannoma have been reported to date^16^ with just few studies concerning the imaging findings of primary adrenal Shwannoma.^1,16–18^ Most of these adrenal tumors is non-functioning (no production of steroid or catecholamine); for this reason it represents a challenging problem for radiologist, that should differentiate it from myelolipoma and other solid non-functioning adrenal tumors.^16^

A correct pre-operative diagnosis of adrenal Schwannoma is essential to avoiding unnecessary extensive surgery and/or regional lymph nodes dissection; in routine clinical practice, the lack of specific clinical and radiological features^19,20^ makes difficult to diagnose adrenal Schwannomas as other malignant tumors of adrenal glands or retroperitoneal organs.^4,8,21^

## Clinical presentation

**Figure 1.**
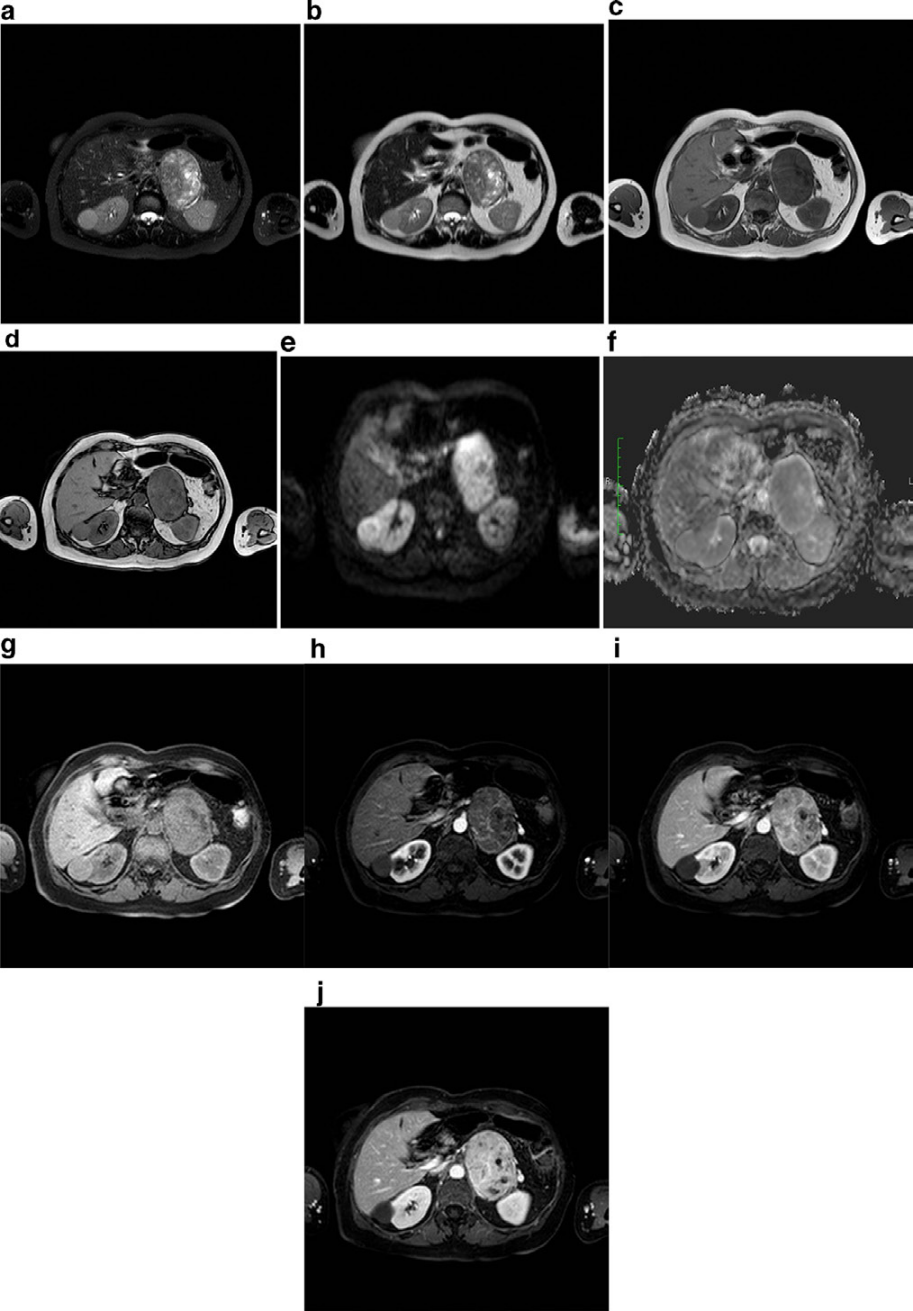
MRI examination of our patient, 61-years-old female, with left adrenal mass. (a) *T*_2_ (fat sat) weighted axial image shows voluminous and well-circumscribed solid mass in the left adrenal gland area, with heterogeneous high signal intensity, due to the presence of cystic components, also visible on (b) *T_2_* weighted axial image. The lesion has well defined margins and fluid signal intensity areas; (c, d) *T*_1_ weighted images in phase and out of phase show the absence of signal intensity drop in opposed phase acquisitions, suggesting that intracellular fat was not present in the mass. Note also the regular profile and smooth margins of the mass; (e, f) DWI b 800 and relative ADC map axial image at the level of the adrenal mass show the presence of moderate diffusivity restriction due to lesion hypercellularity; (g) *T*_1_ (fat sat) weighted images before and (h, i, l) after paramagnetic contrast medium intravenous injection show clearly the progressive and heterogeneous contrast enhancement of the adrenal mass, with no evidence of adjacent structures infiltration. ADC, apparent diffusion coefficient; DWI, diffusion-weighted imaging.

**Figure 2.**
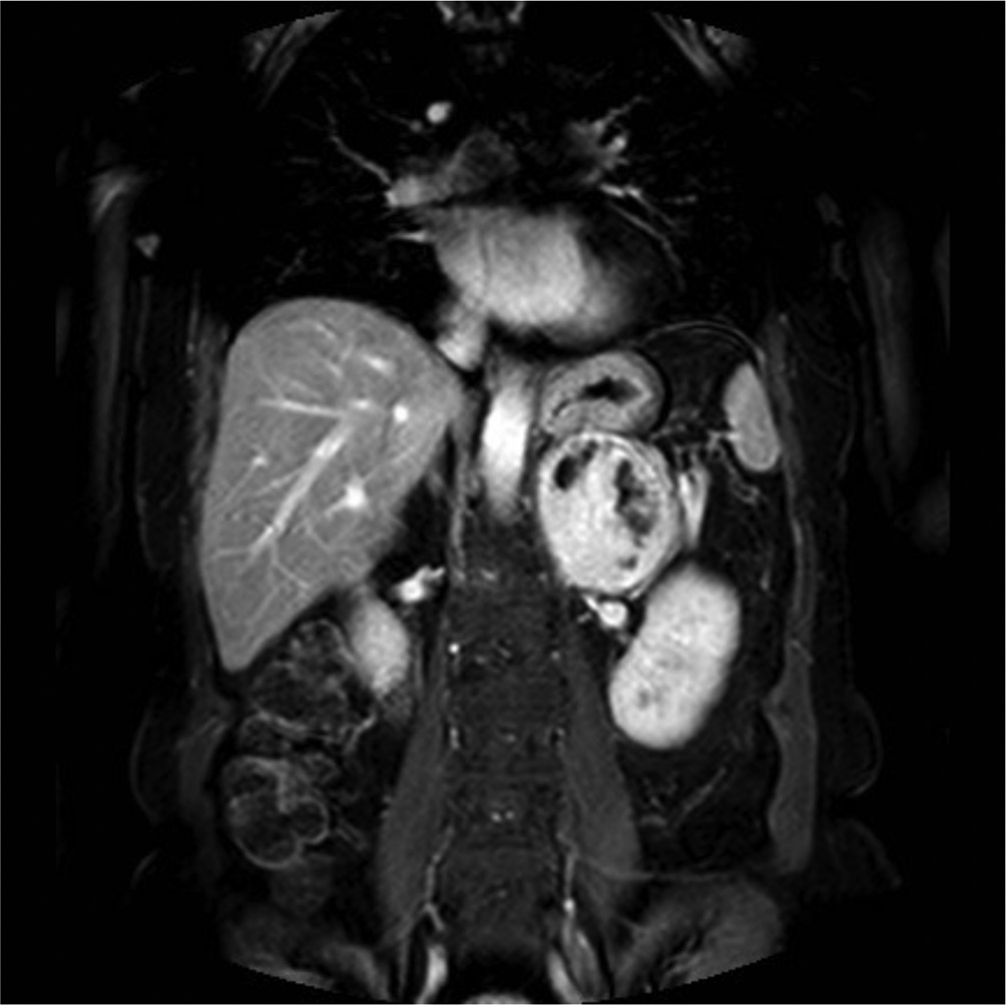
Coronal thrive acquisition after paramagnetic contrast medium intravenous injection in deleyed phase. This plane well displays that mass exhibits close relations with the superior pole of left kidney, stomach, pancreatic tail and left renal vein, aorta and diaphragmatic pillar. There is no evidence of infiltration, as confirmed later by surgery and histology.

**Figure 3.**
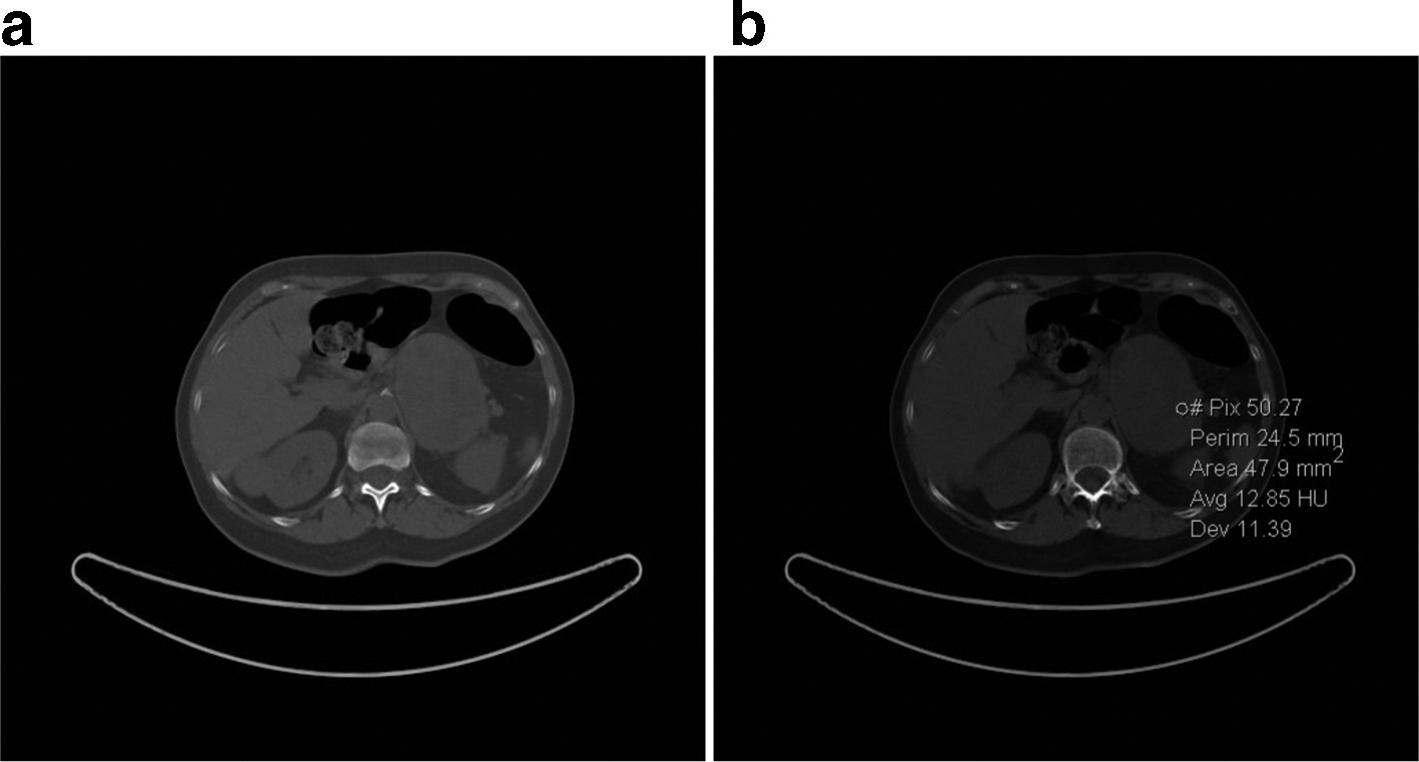
Unenhanced CT axial images show a well-circumscribed heterogeneous mass at the left adrenal level and reveals (a) intraregional cystic-like foci (13 HU) and (b) the absence of calcifications in the mass. HU, Hounsfield unit.

**Figure 4.**
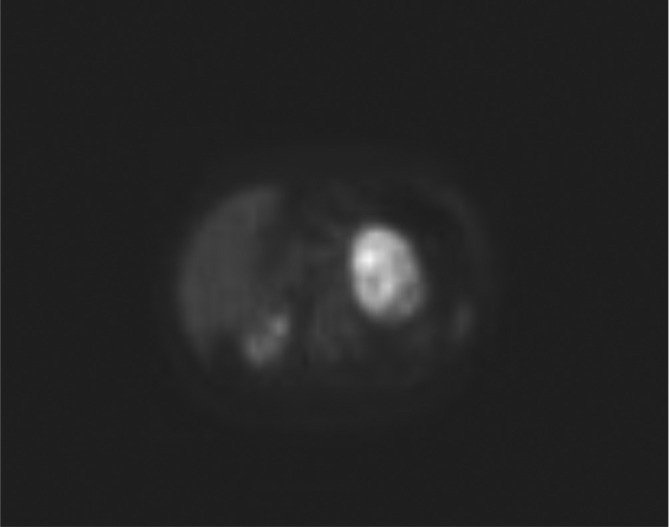
PET-FDG axial image demonstrates heterogeneous and intense FDG uptake in correspondence of the adrenal mass, suggesting a lesion with high metabolic activity. FDG, fludeoxyglucose; PET, positron emission tomography.

**Figure 5.**
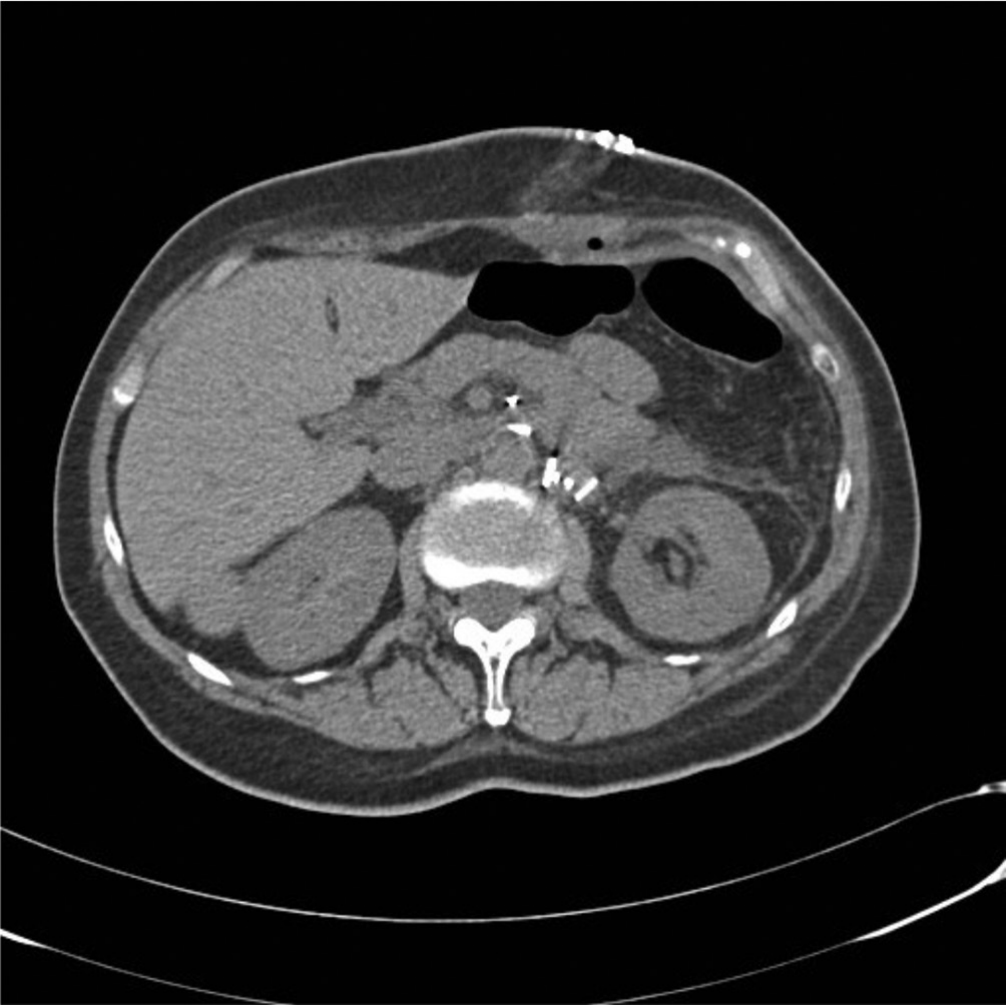
Post-operative unenhanced CT axial image. Surgical clips of left adrenalectomy are present. No injuries of other organ and no fluid or air collection is visible. The patient was discharged on postoperative day 5.

A 61-year-old female with an abdominal mass discovered at abdominal sonography performed for an unrelated reason at another institution was sent to our attention for its characterization. She was asymptomatic and the physical examination was not particularly interesting. Laboratory tests, including serum catecholamines, were within the normal limits. MRI before and after intravenous injection of paramagnetic contrast medium (gadobenate dimeglumine) was performed to characterize the mass: a voluminous solid and heterogeneous mass with fluid components was detected in the left adrenal gland. The mass measured 9 × 6 × 7 cm and showed low signal intensity on *T*_1_ weighted images and heterogeneously high signal intensity on *T*_2_ weighted images, with progressive contrast enhancement after contrast medium injection and without drop of signal in *T*_1_ weighted opposition phase sequence. The tumors showed mild and progressive peripheral enhancement involving especially the wall and the internal septa with irregular pattern due to cystic intralesional components (Figure 1). The adrenal mass was characterized by smooth and regular margins and there was a contact with adjacent structures (superior pole of left kidney posteriorly, bowel loop anteriorly, pancreatic tail and left renal vein inferiorly, aorta and diaphragmatic pillar medially), but without any sign of infiltration (Figure 2). Unenhanced CT, obtained with positron emission tomography-fludeoxyglucose (PET-FDG) study, confirmed the presence of well-circumscribed heterogeneous mass with hypodense foci (<20 HU) in the region of the left adrenal. There was no calcification in the mass (Figure 3). The right adrenal gland was normal. PET-FDG examination showed increased metabolic activity in the region of the mass (Figure 4). After a multidisciplinary evaluation, surgical resection was recommended with a standard transperitoneal adrenalectomy. Intraoperatively there was firm nodular left adrenal mass close to 10 cm, with no infiltration to surrounding tissue. Other organs of the abdomen were normal. Post-operative course was uneventful, and the patient was discharged on postoperative day five (Figure 5). Gross examination of the resected specimen showed a well encapsulated grey white nodular mass (8 cm, 240 gr) with surface showing softening reddish-brown areas and a small flap of non-pathologic fat attached to adrenal gland was also described. Cut section of the mass showed whitish and yellowish areas with faint whorling. Histopathological examination showed a neoplasm composed of cells arranged in interlacing fascicles with alternating hyper- and hypocellular areas. Immunohistochemistry showed positivity for S100 and Ki-67 rate of 1–2% (negativity for alfa-actin, HMB, melan A and CD68). Our patient did not show any familiar tumor syndromes like Neurofibromatosis or Schwannomatosis. A diagnosis of adrenal Schwannoma with degenerative aspects (“ancient Schwannoma”—meaning degenerative alterations characterized by perivascular hyalinization with cystic necrosis, calcification, relative loss of Antoni type A tissue and degenerative nuclei) was made.

## Discussion

Schwannoma is a neurogenic tumor, generally occuring between the third and the sixth decades of life, with no sex or location predilection. In fact, it may arise everywhere in organs or nerve trunks and the with sole exception of cranial nerves I and II, lacking Schwann cells.^13^ Firstly Schwannomas were described by Verocay in 1908 and Antonini in 1920 subclassified these tumors into two distinct histologic patterns.^22^ Typical Schwannoma is a solitary mass, with ovoid or spherical morphology and well-defined margins; when Schwannoma is very large (>8–10 cm) it is frequently affected by degenerative changes with cystic areas, calcifications, interstitial fibrosis and hyalinization. The degenerative pattern is predominant in the “ancient” Schwannoma, a variant with a very good clinical outcome.^23^ It should be known that 5–18% of Schwannomas are associated with von Recklinghausen’s disease; in this pathological context Schwannomas are generally malignant with a trend of manifestation in multiple areas.^24^ The medulla of adrenal gland receives double innervation by two different groups of myelinated nerve fibers, respectively derived from the sympathetic trunk (or vagus nerve) and from the phrenic nerve. Adrenal Shwannomas are thought to origin from one of those nerves and to arise from medulla because of the uninterrupted continuance between the tumor and the adrenal medulla in absence of septum around the tumor. They grow up from adrenomedullary site compressing the adrenal cortex.^16^ Schwannomas present two main microscopic pattern at histological analysis, that are respectively characterized by high cellularity component (areas of Antoni A) and pure myxoid component (areas of Antoni B). Each component may result prevalent in the lesion determining a specular radiological specific findings on CT or MRI scans. Morever, as tumors with neuroectodermal origin, almost all Schwannomas exhibit intense immunohistochemical staining for S-100 protein.^13,25^ A limited number of Shwannomas may be not easily distinguishable from neurofibromas because of their similar histologic aspects and positive staining for S-100 protein.^14^ Moreover, in the assessment of differential diagnosis between these two entities, Fine et al^26^ have demonstrated that a positive stain for calretinin (calcium-binding protein of the same family as S-100) helps for discrimination and allows a diagnosis of Shwannoma. Behavior of these tumors may be biologically benign or not. Malignant Schwannomas are often *ex-novo* lesions, with locally invasive pattern of growth and tendency to develop distant metastases.^25^ On the contrary, benign lesions do not present invasive trend and during expansion they simply displace surrounding structures and organs; however, there is not proven correlation between size and biological malignant potential.^27^ One percent of all retroperitoneal tumors are Schwannomas and only 1–3% of all Schwannomas are located in profound structure such as retroperitoneum and posterior mediastinum, representing uncommon siting of this tumor.^28,29^ Retroperitoneum is a flexible space and Schwannomas detection is often accidental because large size can be reached before they become symptomatic, showing for long time only incipient and vague sign of adjacent organ compression. In advanced stages, space-occupying mass signs manifest with lumbar heaviness or pain, neuralgia and paresthesia in the distribution of the affected nerves, ureteral obstruction; in malignant forms with infiltration of surrounding structures there are generally hematuria and bone pain.^27^ The insufficiency of specific or typical imaging findings on ultrasound, CT or MRI makes the pre-operative diagnosis and the differential diagnosis with other retroperitoneal malignant entity very difficult.^30^ However, the role of radiological imaging is essential in management and treatment planning through examination of tumor size, location, possible invasion of other structures.^13,31^ In accordance with literature, at MRI the Shwannomas show low signal intensity on *T*_1_ weighted images and heterogeneous high signal intensity on *T*_2_ weighted images with cystic areas and smooth profile. However, these imaging characteristics are not specific. Our tumor showed regular margins, heterogeneously high signal intensity on *T*_2_ weighted images, described as necrotic components, and small areas of degeneration on histological examination were present. Most of the head and neck schwannomas are characterized by typical appearance of free diffusion in the centre and restricted diffusion in the periphery of the mass.^32^ In our case, there was not this evidence. Infact, myxoid matrix, abundant in Schwannomas (especially Antoni B tissue within schwannomas), is composed by elevated water content, due to poor cellularity, high mucin and low collagen content, and this likely explain their high diffusivity.^33^ Previous studies described the apparent diffusion coefficient (ADC) values of some adrenal lesions concluding that ADC values could not be used to differentiate between benign and malignant lesions;^34^ in the differential diagnosis between paragangliomas and schwannomas the ADC quantitative assessment could not provide significantly for the differentiation of both tumors.^35^ Classic CT findings of retroperitoneal Schwannomas are rounded and well-marginated masses with various enhancements. Adrenal Schwannomas are hypovascular tumors with mild heterogeneous and delayed progressive enhancement pattern involving equally the tissue, the septa and the walls of the lesion; however, the presence of degenerative changes contributes to make more heterogeneous the enhancement.^31^ The intravenous contrast medium injection should facilitate the differentiation with the adrenocortical carcinoma, characterized by rapid enhancement in hepatic arterial phase, and with pheochromocytoma, which generally is an hypervascular lesion with significant early heterogeneous enhancement.^31^ Calcifications and cystic components are frequently identified in these tumors.^11,12,31^ In our case, unenhanced CT performed during FDG CT-PET examination confirmed the presence of well-circumscribed round mass without evidence of calcifications. Cystic-like areas were visible and also detected trough small region of interest placement. The mass showed also an increased FDG uptake. Non-functioning solid tumors represent the main differential diagnoses of adrenal Schwannoma, which is not easily distinguishable from adrenal adenoma, adrenal myelolipoma, groups of neuroblastomas (neuroblastoma, ganglioneuroma, ganglioneuroblastoma), adrenocortical carcinoma and adrenal metastasis. Adrenal adenomas and adrenal myelolipomas generally have a fat content that makes possible to differentiate them from other tumors. Neuroblastomas are positive during metaiodobenzylguanidine (MIBG) scintigraphy in seventy percent of cases and, among the neuroblastomas, ganglioneuromas are similar to retroperitoneal Schwannomas on imaging studies. However, although MIBG-negative ganglioneuromas are difficult to differentiate from Schwannomas^36^ , Ichikawa et al() described delayed heterogeneous enhancement of ganglioneuroma on contrast- enhanced CT and whorled appearance with curvilinear bands of low signal intensity on *T*_2_ weighted images on MRI.^36^ Radiologist should keep in mind these findings that could be helpful in the differential diagnosis between MIBG-negative ganglioneuromas and other tumors. In our experience, the preoperative diagnosis was erroneous and misinterpreted the nature of the lesion, suggesting the possibility to be facing an adrenal ganglioneuroma or pheochromocytoma. Our experience confirms the difficulties in obtaining a correct diagnosis before pathological examination.

## Learning points

- In summary, primary adrenal Schwannomas are rare and difficult to diagnose and frequently pre-operative workup is non-diagnostic, only postulating a non-secreting adrenal mass.
- For this reason, adrenal Schwannomas should be always included in the differential diagnosis of solid nonfunctioning adrenal tumors.
- MRI is a valuable imaging technique for the diagnosis of adrenal Schwannoma, which appears as a well-circumscribed unilateral mass with cystic degenerative changes, characterized by low signal intensity on *T*_1_ weighted images and heterogeneous high signal intensity on *T*_2_ weighted images.
- As confirmed by literature and by our experience, radical excision must be considered the most appropriate treatment, resulting in satisfactory prognosis.
- Given the rarity of this tumor and lack of definitive nonhistologic diagnostic modalities, adrenal Schwannoma remains a diagnosis of exclusion, but it should occupy an important place in the differential diagnosis set of incidentally discovered adrenal masses, as illustrated by this case.
